# Nanoscale MRI for Selective Labeling and Localized Free Radical Measurements in the Acrosomes of Single Sperm Cells

**DOI:** 10.1021/acsnano.2c02511

**Published:** 2022-06-30

**Authors:** Claudia Reyes-San-Martin, Thamir Hamoh, Yue Zhang, Lotte Berendse, Carline Klijn, Runrun Li, Arturo E. Llumbet, Alina Sigaeva, Jakub Kawałko, Aldona Mzyk, Romana Schirhagl

**Affiliations:** † Groningen University, University Medical Center Groningen, Antonius Deusinglaan 1, 9713 AW Groningen, The Netherlands; ‡ Laboratory of Genomics of Germ Cells, Biomedical Sciences Institute, Faculty of Medicine, University of Chile, Independencia, 1027, Independencia, Santiago 8380000, Chile; § AGH University of Science and Technology, Academic Centre for Materials and Nanotechnology, Al. A. Mickiewicza 30, 30-059 Krakow, Poland; ∥ Institute of Metallurgy and Materials Science, Polish Academy of Sciences, Reymonta 25, 30-059 Krakow, Poland

**Keywords:** NV centers, Nanodiamonds, Relaxometry, Free radicals, Sperm cells, Infertility

## Abstract

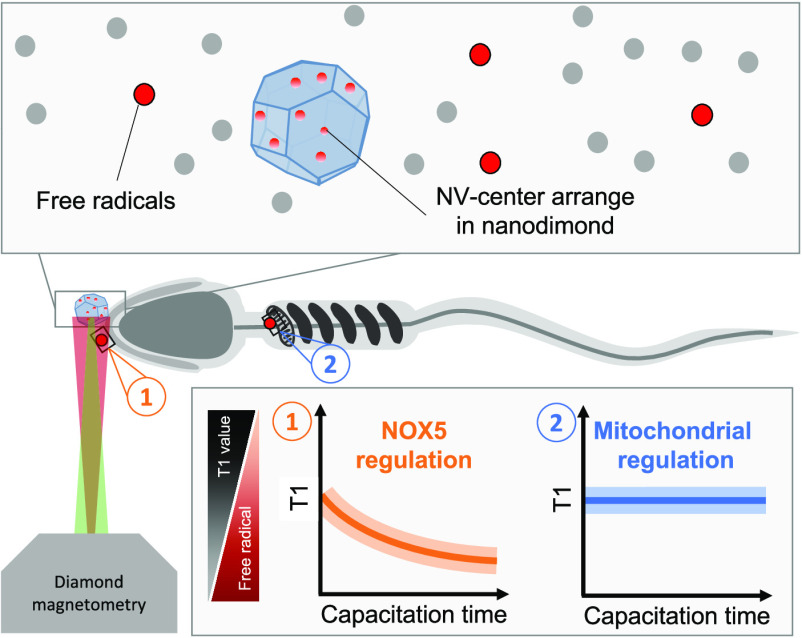

Free radicals play a major role in sperm development, including maturation and fertilization, but they are also linked to infertility. Since they are short-lived and reactive, they are challenging to detect with state of the art methodologies. Thus, many details surrounding their role remain unknown. One unknown factor is the source of radicals that plays a role in the sperm maturation process. Two alternative sources have been postulated: First, the NADPH-oxidase system embedded in the plasma membrane (NOX5) and second, the NADH-dependent oxidoreductase of mitochondria. Due to a lack of localized measurements, the relative contribution of each source for capacitation remains unknown. To answer this question, we use a technique called diamond magnetometry, which allows nanoscale MRI to perform localized free radical detection. With this tool, we were able to quantify radical formation in the acrosome of sperm heads. This allowed us to quantify radical formation locally in real time during capacitation. We further investigated how different inhibitors or triggers alter the radical generation. We were able to identify NOX5 as the prominent source of radical generation in capacitation while the NADH-dependent oxidoreductase of mitochondria seems to play a smaller role.

## 1 Introduction

The free radical theory of infertility is derived from the more general free radical theory of aging. It states that free radicals are the reason why sperm cells become dysfunctional. As the free radical theory of aging itself, it is intensely debated. Additionally, free radicals or oxidative stress are expected to be linked to various pathogenic conditions that impact male fertility.^1−3^ However, some reactive oxygen species are also needed to maintain crucial functions in sperm cells including sperm capacitation, the acrosome reaction, and sperm-oocyte fusion.^4,5^

Several techniques have been used to measure free radicals in cells. There are indirect and direct methods. Indirect methods measure the response from or damage to the cell rather than the radicals themselves. This can be achieved for example by quantifying enzymes, which are responsible for degrading radicals. The advantage of these techniques is that they are specific for certain radicals. In sperm cells, for instance, superoxide dismutase activity indicates the presence of superoxide radicals and is correlated with sperm concentration and overall motility.^6^ Another study showed that glutathione peroxidase^7^ was reduced in patients with certain sperm dysfunctions. However, such techniques require prior knowledge on which radicals are produced and which enzymes the cells use to cope with the radical load. Additionally, these methods do not reveal the location where free radicals were generated. On the other hand, direct methods rely on compounds that react with the radicals and are converted into a fluorescent or chemiluminescent form.^8^ Most compounds are nonspecific and generally react with all kinds of reactive species. However, there are also some more specific probes. All fluorescent compounds measure the amount of radicals or reactive oxygen species (ROS) that have been created between adding the compound and the measurement. Due to bleaching it is usually only possible to do a one-time measurement. For sperm cells there are also several of this kind of probes that have been used successfully. One of the most commonly applied probes, 2,7-dichlorofluorescein diacetate (H_2_DCFDA), reacts with multiple different ROS.^9^ Dihydroethidium or hydroethidine (DHE), for instance, are specific for O_2_^•–^, and hydrocyanine dyes are specifically designed for O_2_^•–^ and OH^•^.^10,11^

Diamond magnetometry potentially offers a complementary solution. This technique is based on defects in diamonds, which change their optical properties based on their magnetic surrounding. Since optical signals can be read out more sensitively, this method offers nanoscale magnetic resonance signals high sensitivity.^12−14^ So far, this technique has been successfully used for measurement of magnetic vortices, nanoparticles, or molecules on the diamond surface.^15−17^ Arguably, the most impressive achievements with the technique are measurements of single electrons or even a few nuclear spins.^18−20^ In a biological environment, several measurements have been done already as well. Ermakova et al. showed the detection of iron in ferritin.^21^ Several groups were able to measure either spin labeled cells or magnetic particles in a cellular environment.^16,22,23^ Relaxometry, a specific mode of diamond magnetometry, is particularly useful due to its high sensitivity to spin noise.^24^ This measuring mode is also very convenient since both spin manipulation and read out are purely optical and there are no microwaves required. Also this method has been used already successfully in several different applications.^25−28^ Since free radicals have a free electron spin, they cause a spin noise, which can be measured with this technique.^12−14^ Most recently, our group has demonstrated that the technique is suitable for nanoscale detection of free radicals in living cells. This has been shown for metabolic activity in immune cells as well as aging in yeast cells.^29,30^ Here we show that this technique can be used to measure free radical generation from sperm cells in real-time. We also shed light on the role of the NOX5 and mitochondrial NADH-dependent oxidoreductase in free radical-dependent capacitation and the mechanism of action of progesterone.

## 2 Results and Discussion

### 2.1 Fluorescent Nanodiamonds (FNDs) Are Preferentially Localized on the Head of Sperm

**Figure 1:**
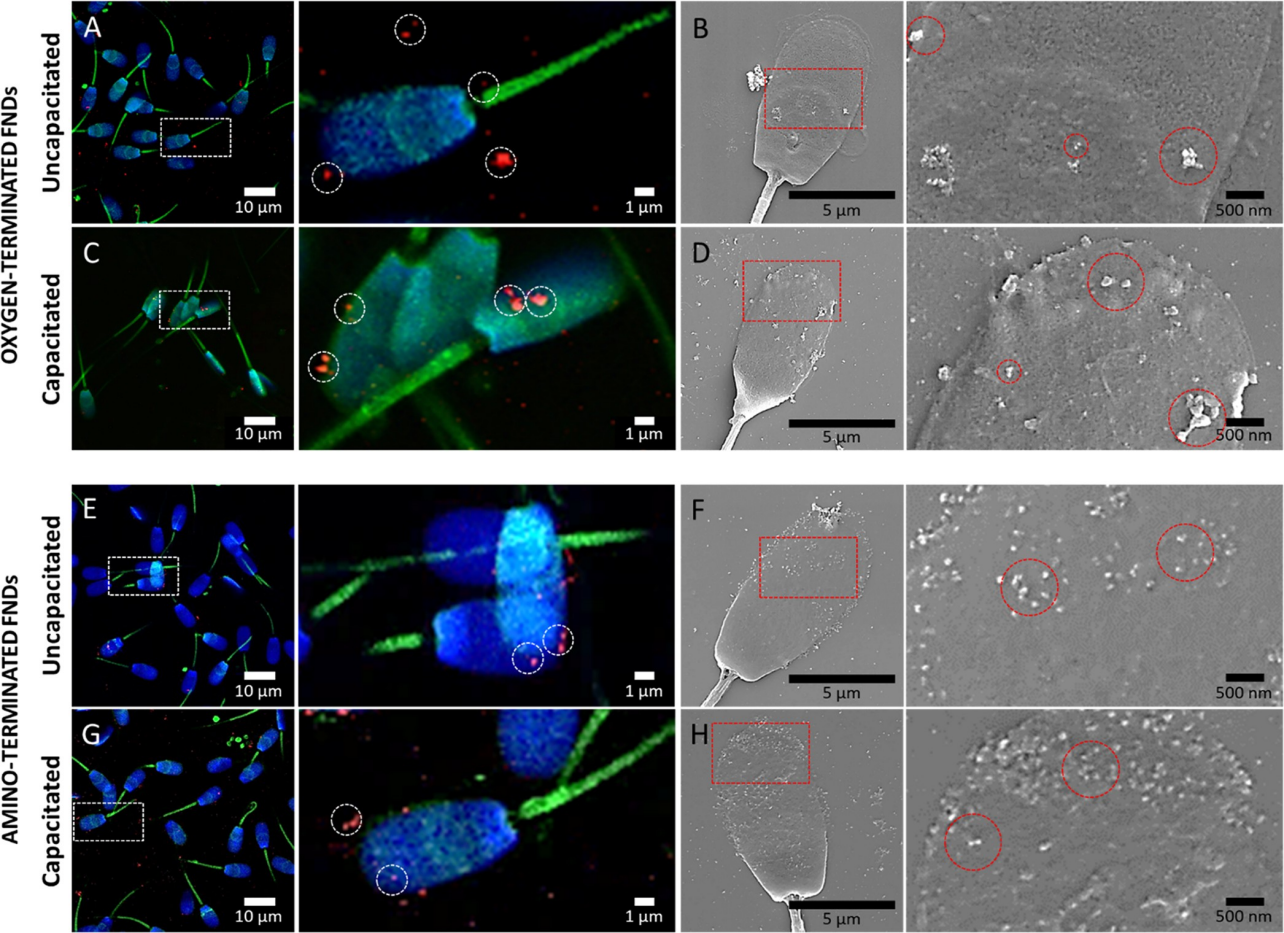
Localization and morphology of fluorescent nanodiamonds in sperm cells. We observed confocal (left panel) and SEM (right panel) images of sperm cells incubated with either oxygen-terminated (A-D) or amino-terminated (E-H) FNDs at 1 μg/mL. Two capacitation statuses were evaluated: uncapacitated (A, B, E, F) and capacitated (C, D, G, H). The dashed circles indicate a few nanodiamonds to give an example. Confocal images show in green, phalloidin-FITC labeling F actin filaments; blue, DAPI labeling the nuclei; red, FNDs. The samples were imaged using 405, 488, and 561 nm lasers of a Zeiss LSM780 confocal microscope. SEM images were acquired in a FEI Versa 3D FEG scanning electron microscope using an acceleration voltage of 10 kV and a beam current of 4 nA.

Treatment of immobilized sperm cells with oxygen-terminated or amino-terminated FNDs resulted in particles preferential attachment to the spermatozoa head (Figure 1). Based on scanning electron microscopy (SEM) images, we distinguished two populations of sperm cells with different midpiece morphology (Figure S1). We found that FNDs do not attach to the sperm cells with the smooth midpiece. The amino-terminated nanodiamonds had a higher affinity for the membrane of sperm cells and formed smaller aggregates than the oxygen-terminated variants. The capacitation process increased the number of attached particles.

### 2.2 FNDs Are Not Affecting Viability and Metabolic Activity of Sperm Cells

**Figure 2:**
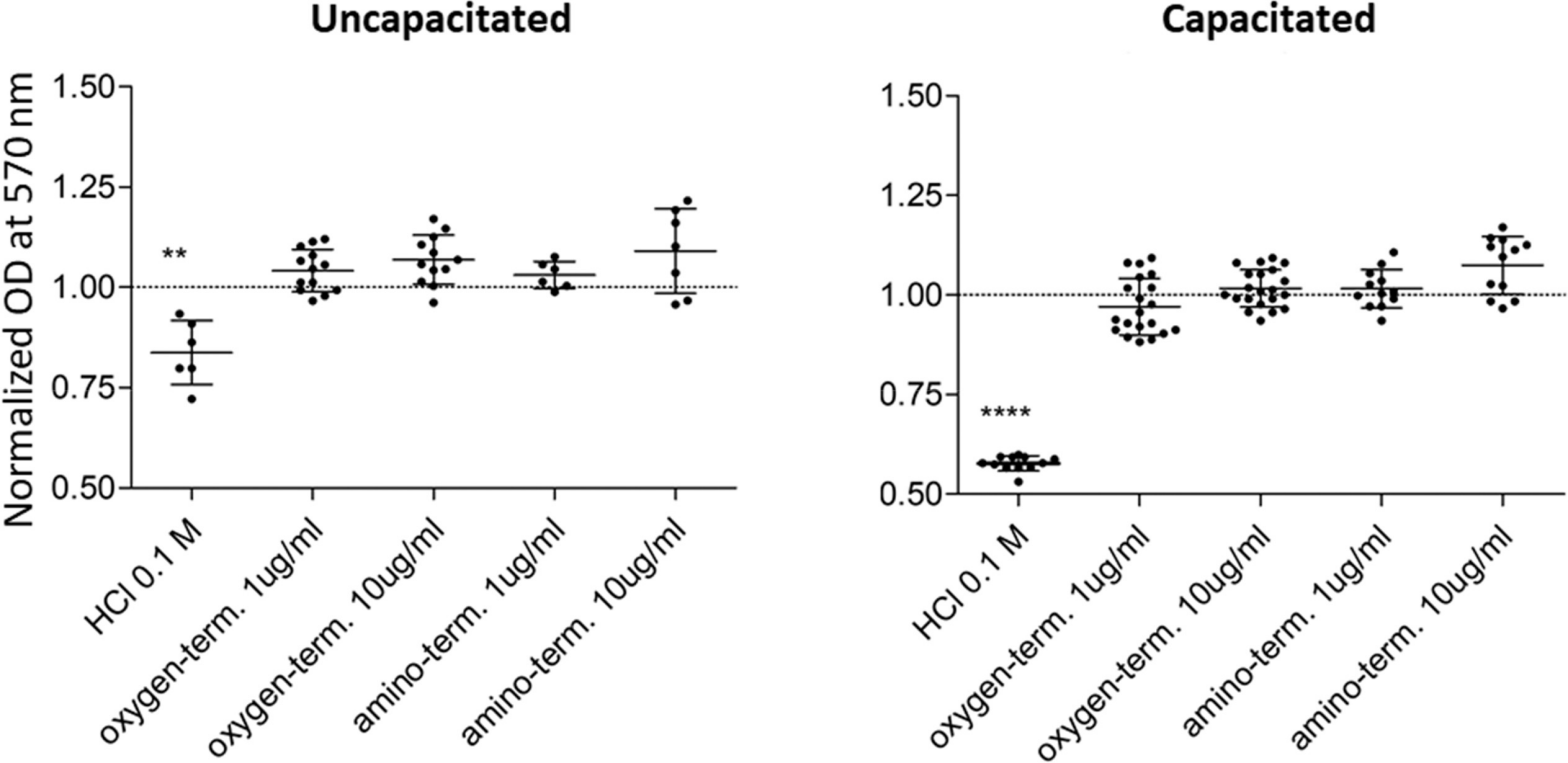
Metabolic activity of sperm cells determined via a thiazolyl blue tetrazolium bromide (MTT) assay. The optical density indicates the sperm cell’s capability to metabolize MTT to formazan at 570 nm. Sperm cells were treated with the oxygen-terminated or amino-terminated fluorescent nanodiamonds. Results were normalized in accordance to control untreated sperm cells.

**Figure 3:**
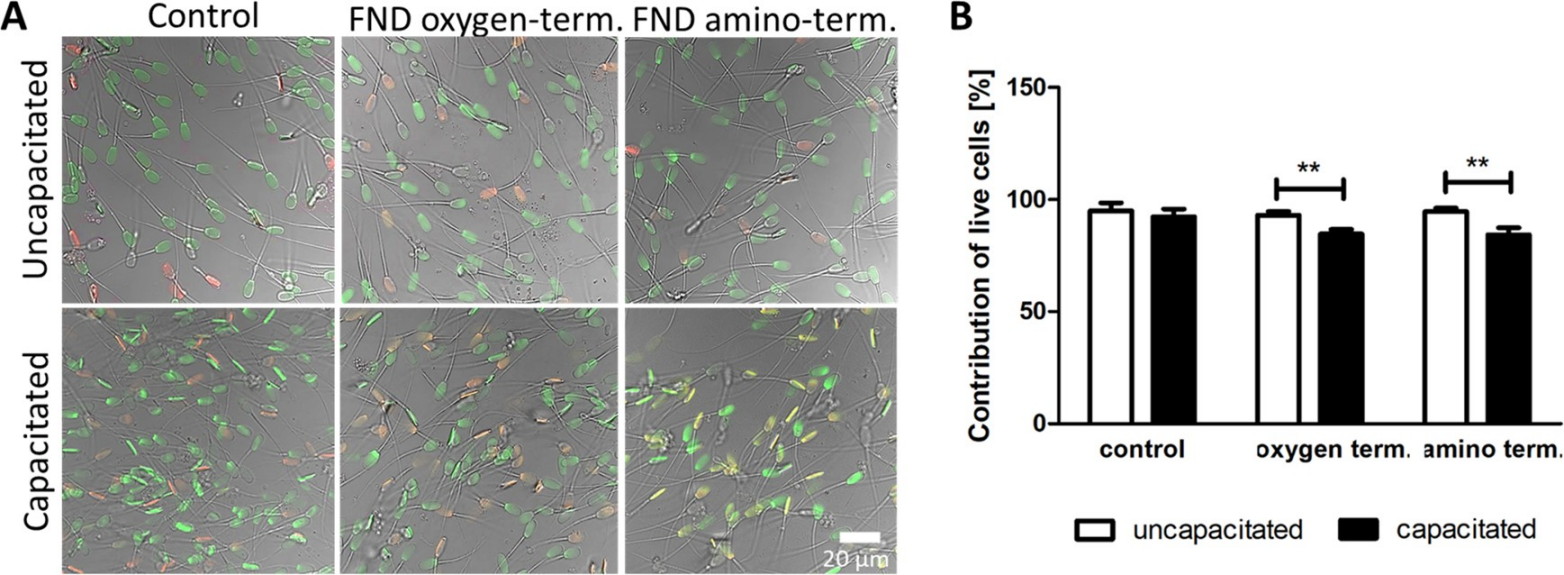
Membrane integrity of uncapacitated and capacitated sperm cells treated with 1 μg/mL oxygen- or amino-terminated FNDs. (A) Representative images from the life-dead staining (SYBR-14 staining living cells in green and propidium iodide staining cells with damaged membranes in red) and (B) quantitative assessment where 300 cells were counted.

The metabolic activity of spermatozoa was not affected by treatment with amino- or oxygen-terminated nanodiamonds (Figure 2). We did not observe any adverse effects even when FNDs were used in a concentration that is relatively high for magnetometry applications (10 μg/mL). There was no difference between metabolic activity of the uncapacitated and capacitated sperm cells. We also noticed a slight impact of either oxygen- or amino-terminated FNDs (1 μg/mL) on sperm membrane integrity. This is shown in the LIFE/DEAD assay in Figure 3. The number of live, green-stained cells was slightly lower for the capacitated state due to membrane permeability changes. For each FND variant, live cells were more abundant than dead, red-stained sperm cells.

### 2.3 Oxygen-Terminated FNDs Are Suitable for Real-Time Free Radical Detection in Sperm Cells

**Figure 4:**
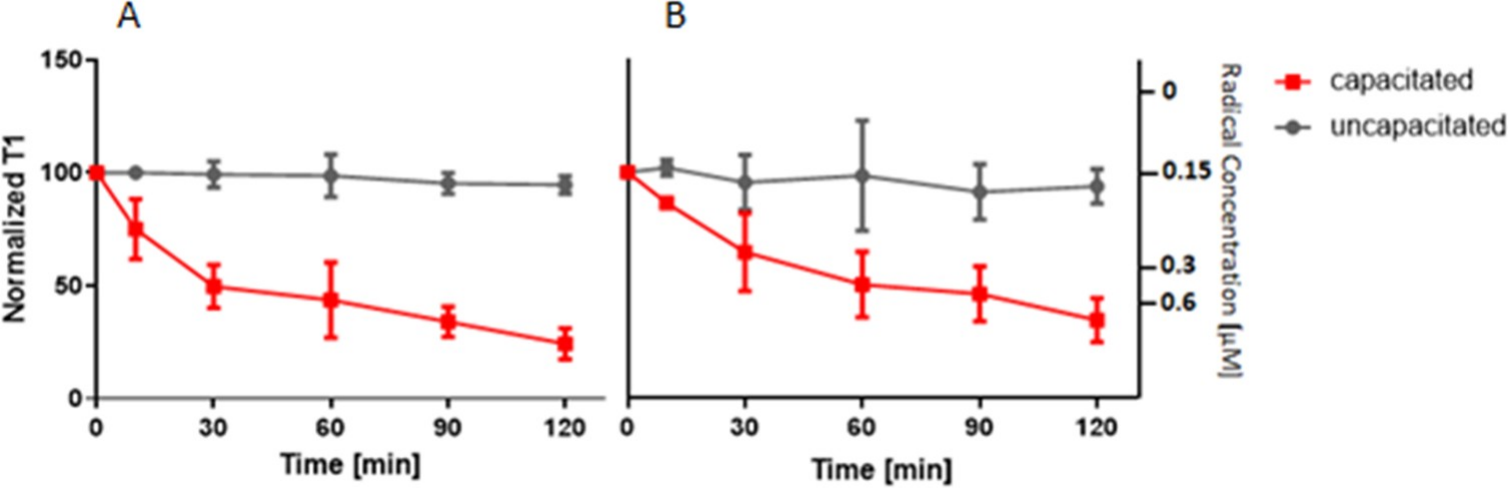
Free radical generation during sperm capacitation. Results were obtained using (A) oxygen-terminated and (B) amino-terminated nanodiamonds. We plot the normalized T_1_ response (left *y*-axis). As an approximation, we plot the concentration of radical formation (right *y*-axis) determined from a calibration curve with *OH radicals in solution from our previous work.^26^ Error bars represent the standard error of the mean, while each time point represents a measurement of three experiments.

Both types of nanodiamonds were used in relaxometry measurements to detect real-time changes in free radical levels during the sperm transition from the uncapacitated to the capacitated state (Figure 4). We tracked the capacitation progress for 2 h using one selected nanodiamond on the acrosomal part of a motile sperm cell (Figure S2, video) and observed gradual shortening of the relaxation time. A decrease of T_1_ values corresponds to an increase in free radical concentration near the nanodiamond sensor, which was expected for the capacitation process. We did not observe any significant changes of T_1_ values over 2 h measurements for the control samples in which we just refreshed the noncapacitating medium. At the same time, a decrease in T_1_ was recorded for both FND variants. For the results obtained using amino-terminated particles, we observed larger variabilities than for oxygen-terminated FNDs. Since we also observed this in the control, we attribute the larger variability to a greater variability in the NV^–^ centers rather than biological variability.

### 2.4 Relaxometry Reveals Localized Changes in the Kinetics of Capacitation

**Figure 5:**
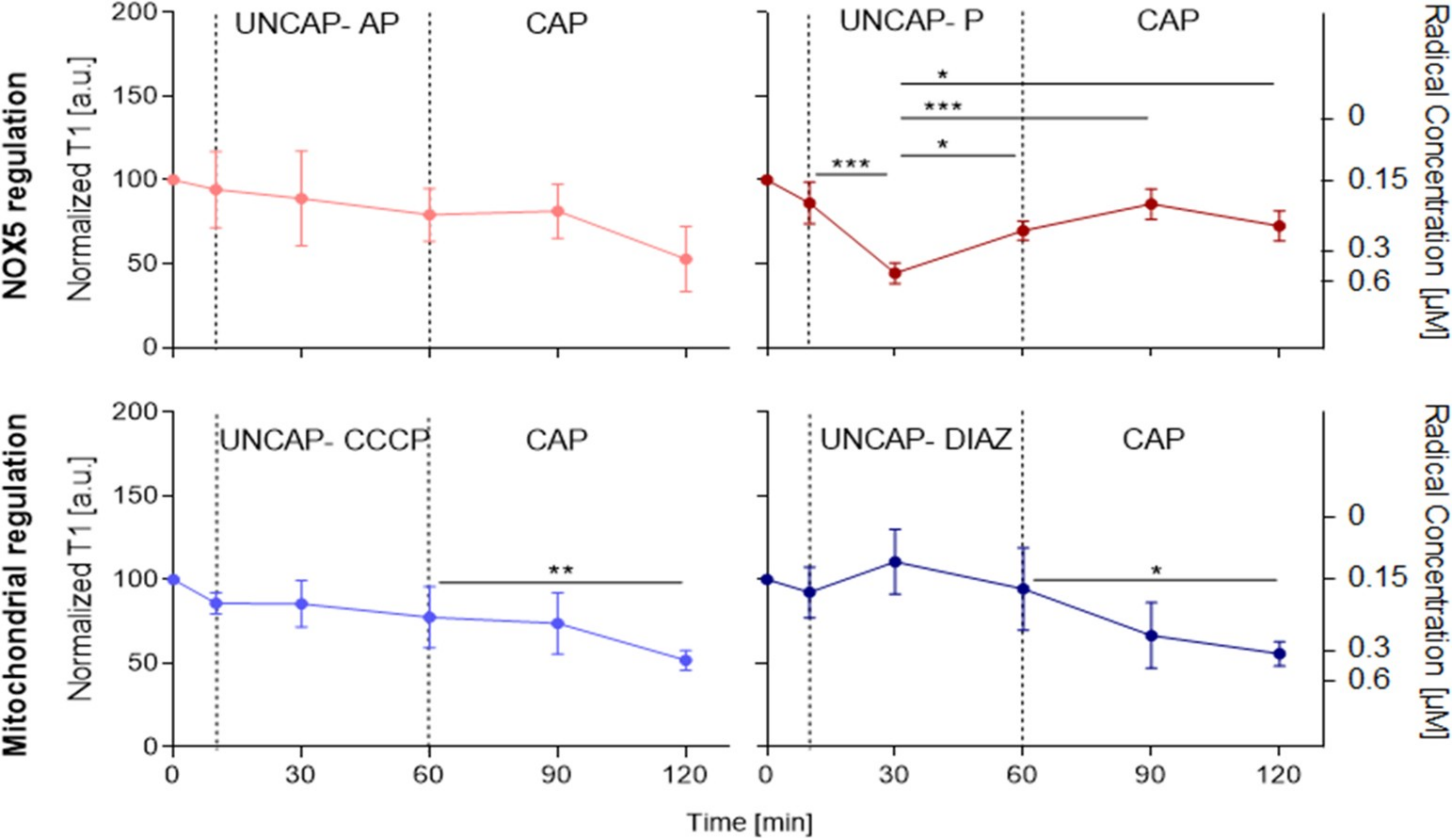
Free radical detection in single acrosomes in order to track kinetics of capacitation in response to the selected triggers and inhibitors. We plot the normalized T_1_ response (left *y*-axis) and an approximation of the concentration of radical formation (right *y*-axis) as a function of time. Error bars represent standard deviation, while each time point represents a measurement of six experiments.

In further experiments, only the oxygen-terminated FNDs have been used due to better sensing performance (smaller variability). The uncapacitated sperm cells were incubated with FNDs and subsequently treated with agents that inhibit radical generation in the defined cell areas (mitochondria or acrosome). The activity of NOX5 in the plasma membrane of the sperm head was blocked by apocynin (AP in pink) and then cells were subjected to capacitation (Figure 5). The concentration of free radicals did not significantly change when the inhibitor was added to uncapacitated sperm cells and remained on the same level after capacitation. The inhibitory effect of apocynin persisted for 1 h.

Complementary, we have inhibited mitochondria activity with *m*-chlorophenylhydrazone (CCCP) (in sky blue) followed by the capacitation process. The mitochondrial inhibitor did not have a significant impact on the free radical level in uncapacitated sperm cells. Nevertheless, we have observed an increase in the free radical concentration after capacitation. This proves that relaxometry enables localized detection of radicals generated by NOX5 in sperm heads.

Besides the effect of inhibitors, we investigated how the addition of activators of free radical generation either by the NOX5 (progesterone, P in dark red) or mitochondria (diazoxide, DIAZ in dark blue) influences the kinetics of the capacitation process. Progesterone enhances capacitation via opening calcium specific channels. This promotes cellular uptake of calcium ions required for NOX5-dependent generation of free radicals. Surprisingly, we have found that progesterone had a significant impact on free radical generation in sperm cells incubated in an uncapacitating medium that did not contain calcium. We reported a temporary increase in free radical concentration followed by a process of a gradual return to the initial status. Then sperm cells were subjected to capacitation medium with calcium (HTF). We did not observe any significant change in free radical generation within 1 h. Contrarily, we did not register any significant change in T1 values when cells were treated with diazoxide, which is acting as inhibitor of the mitochondrial complex II of the electron chain to stimulate the formation of free radicals on the Q site of complex III.^36^ Nevertheless, the free radical concentration increased after capacitation.

**Figure 6:**
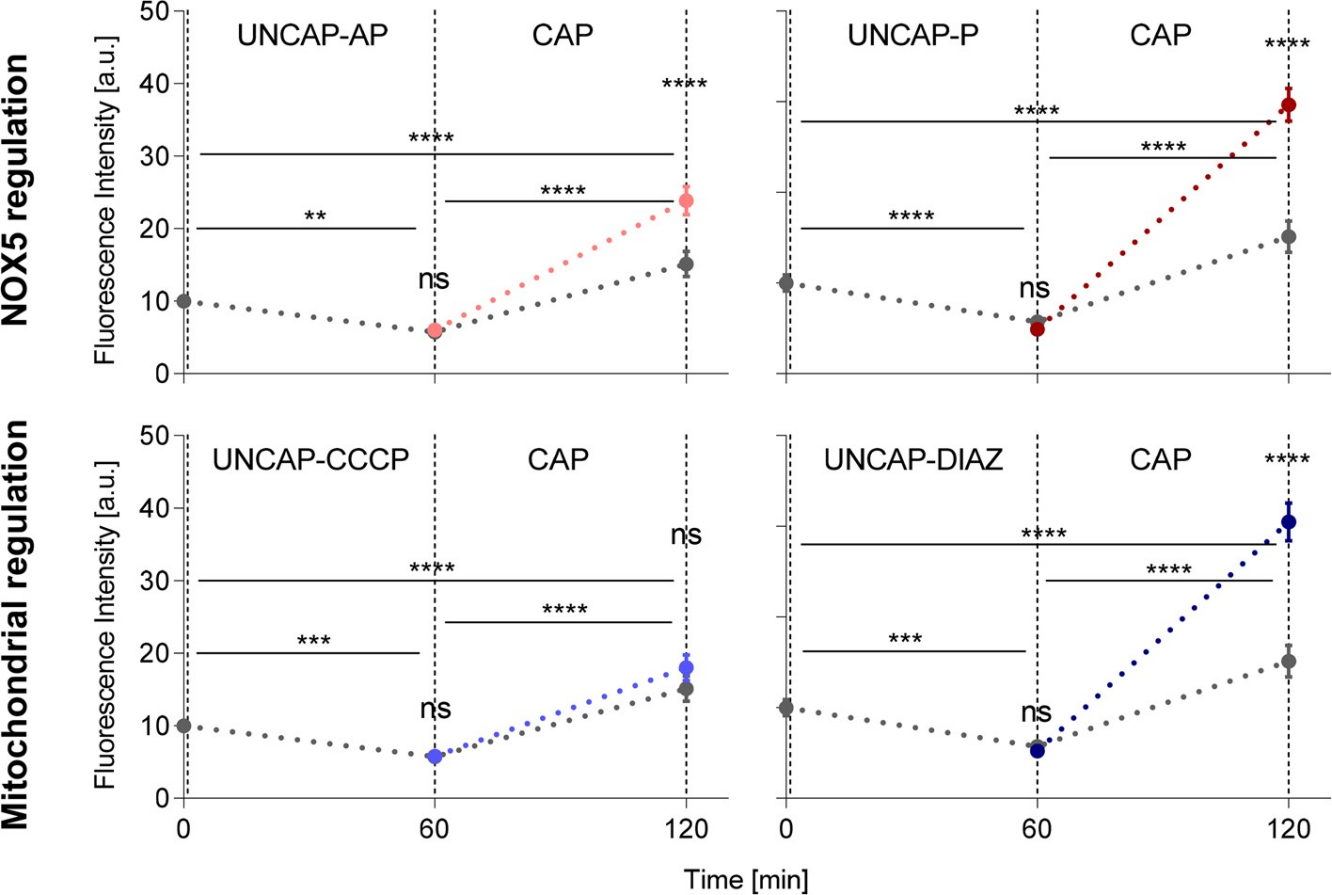
Cellular superoxide detection in the head of sperm cells in response to selected stimuli. Inhibitors: apocynin, AP in pink, and carbonyl cyanide-chlorophenylhydrazone, CCCP in sky blue; and triggers: progesterone, P in dark red, and diazoxide, DIAZ in dark blue. We plot the fluorescence intensity at 620 nm in the head of sperm cells at 60 and 120 min after the beginning of the treatment. The control in gray corresponds to changes in capacitating medium mHTF-mHTF-HTF. Error bars represent confidence interval at 95%, a total 3000 cells were analyzed.

In parallel, we measured the superoxide production in sperm cells under capacitation conditions and their regulation at NOX5 and mitochondria level (Figure 6). The fluorescence intensity at 620 nm was measured in the heads of sperm cells at three different time points: 0, 1, and 2 h. Where 0 h corresponds to sperm cells in an uncapacitating medium, 1 h means after 1 h of treatment with the chemical agent (AP in pink, P in dark red, CCCP in sky blue, DIAZ in dark blue, and mHTF only control in gray), and 2 h means 1 h with the chemical agent followed by 1 h in capacitating medium (HTF). We observed a decay in the fluorescence intensity, meaning lower concentration of superoxide, after 1 h in the mHTF medium for control samples, followed by a significant increase after treatment with the HTF medium. The same trend is shown for all chemical agents used. However, the level of increase at the 2 h time point is higher compared to the controls when activators of NOX5 (P) and mitochondria (DIAZ) were used, while inhibitors of mitochondria (CCCP) did not show any significant difference. Surprisingly, the use of AP showed a boost in superoxide production at 2 h compared to the controls.

**Figure 7:**
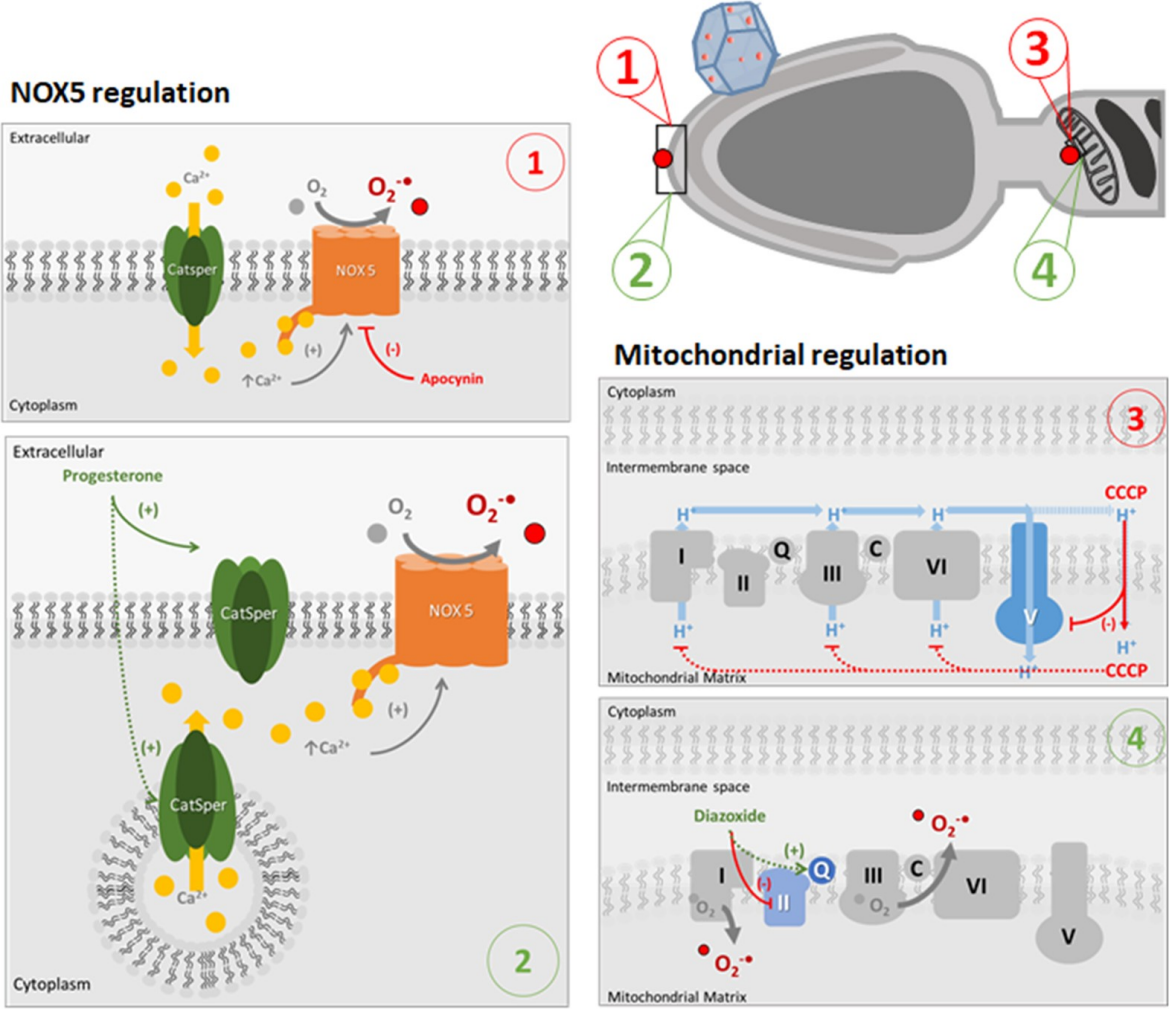
Schematic representation of free radical production in sperm cells and drug treatments. Free radicals in sperm are produced in two main sources:^4^ cell membrane in the acrosome (indicated with labels 1 and 2) and mitochondria (labels 3 and 4). Normally, in the presence of capacitation factors, calcium ions go through calcium channels (CatSper) and bind NOX5, which produce superoxide radicals. Apocynin acts as an inhibitor of NOX5, as a result a decrease in the superoxide production is seen (label 1). In label 2, an alternative pathway for NOX5-dependent free radical production under uncapacitating conditions is presented. Under uncapacitating conditions, there are no calcium ions in the culture medium. However, the presence of calcium ion reservoirs in vesicles containing CatSper channels in the acrosome has been previously reported.^41,42^ We propose that progesterone can activate intracellular CatSper channels allowing the release of calcium ions to the cytosol and therefore activate NOX5 and increase free radical production under uncapacitating conditions.

FNDs are biocompatible in many mammalian cells while combining exceptional biolabeling and sensing properties.^19,37^ In this article, we have shown that fluorescent nanodiamonds can detect free radical generation of a single sperm cell in real time (Figure 7). It has to be noted that at this time we are limited to sensing at the surface since particles do not enter the cells. This is a limitation for applications where radicals need to be detected deeply inside sperm cells. This current limitation might be overcome in the future by altering the surface chemistry or the permeability of sperm cells. We found that FNDs attach only to the subpopulation of mature sperm cells with visible helical mitochondria sheaths. Immature sperm cells carry a cytoplasmic droplet in the neck when they are released into the lumen of seminiferous tubules. This droplet is eliminated in the epididymis, where the maturation process occurs from caput to cauda. Cytoplasmic droplet removal is accompanied by a change in the morphology of sperm midpiece from smooth to helical, which corresponds to the rearrangement of mitochondria that change from round-shaped toward elongated organelles wrapping around the sperm midpiece. Importantly, besides droplet elimination, another important process occurring during epididymal maturation is the removal and adsorption of peripheral proteins on the sperm surface. Gaining a new set of proteins on their surfaces is crucial for sperm cells to fertilize since proteins produced in the epithelial lining of the epididymis are involved in zona pellucida recognition.^38−40^ We think that this shifting in the set of peripheral proteins on the sperm cells’ surface can be related to the fact that FNDs were only able to attach to mature boar sperms. Sperm cells have a net negative membrane charge, but during their epididymal maturation, the dynamic process of peripheral proteins exchange may confer a positive charge to some areas of the sperm’s membrane. In this article, sperm cells were treated with oxygen- or amino-terminated FNDs characterized by a net negative and positive zeta potential, respectively. We found that both types of FNDs attach only at the acrosome of the sperm cell, regardless of their zeta potential. This suggests the presence of positively charged regions on the membrane of sperm heads. Moreover, there was a relation between the surface chemistry and the number of attached FNDs. We observed that significantly more positively charged FNDs adhere to the sperm cells than negatively charged oxygen-terminated nanoparticles. This disproportion slightly decreased after capacitation. It could be an effect of increased positive net charge of the cell surface due to membrane reorganization and removal of glycoproteins as described by Tecle and Gagneux. Magdanz et al. have shown that reduced adsorption of negatively charged silica particles occurred upon maturation of the sperm cells. At the same time, they did not observe any changes in the binding of positively charged iron oxide particles.^40^ A high number of amino-terminated FNDs attached very close to each other at the membrane. This deteriorated sensing performance compared to the oxygen-terminated FNDs, which bind as single and relatively small aggregates. It is worth highlighting that neither abundance nor different surface chemistry of FNDs has affected sperm cells viability, metabolic activity, or redox potential. These studies were a continuation of our previous work where we have shown that the oxygen-terminated 70 nm FNDs at low concentrations can be used for fluorescent labeling of sperm cells due to their biocompatibility. Herein, we prove that FNDs can serve as nanoscale resolution sensors for free radicals during capacitation. Capacitation is a dynamic process that involves a sequence of molecular changes and is required for the successful interaction of sperm cells with oocytes. The current understanding of the biochemical pathways linking free radicals with capacitation still contains many gaps. The exact sources of the free radical generation in sperm cells are subject to discussion. Agarwal et al. indicated two major ways spermatozoa may generate free radicals: (1) The NADPH-oxidase system embedded in the plasma membrane (NOX5) and (2) NADH-dependent oxidoreductase of mitochondria. However, the relative contribution of each source for capacitation remains unknown. The reason is that current methodologies cannot differentiate between these locations. We attempt to monitor the generation of free radicals in real-time, in sperm cells during capacitation. We have demonstrated that T_1_ relaxometry enables the detection of local changes. We have observed a gradual increase of the free radical load during capacitation in the presence of a medium containing albumin, calcium ions, and bicarbonates. Results obtained from experiments with inhibitors (apocynin and CCCP) suggest that free radicals were generated locally by NOX5 in the head of sperm cells. In the next step, we triggered mitochondrial generation of free radicals with diazoxide. The results of this experiment confirmed previous observations. We have not observed any changes in the signal measured in the head region of sperm cells. Therefore, we can conclude that free radicals generated by mitochondria do not take a part in the capacitation process or their contribution is negligible.

Additionally, we activated the NOX5-based free radical generation. We applied progesterone in order to induce calcium uptake by sperm cells through the opening of the specific CatSper channels. We expected an increase in the free radical generation or a more rapid NOX5 response when calcium ions were provided with the capacitation medium. Unexpectedly, the response was observed in the absence of an external source of calcium in uncapacitating conditions. We did not find any time dependent changes in the free radical load in the same medium without progesterone. This phenomenon was not described until now in the literature. We postulate that progesterone acts on CatSper channels localized in the head region of sperm cells. These channels are potentially gating efflux of the intracellular calcium ions stored in cytoplasmic droplets or the acrosome or postacrosome membrane domains. Carrillo et al. proved the presence of CatSper channels,^42^ whereas Correia et al. postulated the existence of calcium storage in the mentioned areas.^43^ The outcome of our study sheds light on progesterone-mediated capacitation.

Furthermore, our proof-of-concept work offers a perspective to explore unknown aspects of capacitation, bringing the potential for testing newly developed drugs or tracking the effects of chemotherapy. It will also be fascinating to determine whether the decreased seminal antioxidant protection observed in cases of male infertility is a result of local or systemic pro-oxidant factors. Finally, further development of diamond magnetometry and its spectroscopic abilities might allow us to distinguish what type of free radicals are involved in specific processes.

The second source is mitochondria (label 3), which produce superoxide radicals as a subproduct of the electron transport chain. CCCP is an uncoupler of the electron transport chain that binds protons in the intermembrane space and carries them to the mitochondrial matrix, inhibiting the action of complex V and paralyzing the free radical production. Label 4 depicts the stimulation of free radicals production by diazoxide on the Q site of the complex III of the mitochondrial electron chain.^36^

## 3 Conclusions

In this work, we used relaxometry to study the dependency of the capacitation process in boar sperm cells on NOX5 or mitochondria. As diamond magnetometry offers localized measurement, we were able to identify that NOX5 plays the main role in the capacitation process and mitochondria has smaller influence.

## 4 Experimental Methods

### 4.1 Fluorescent Nanodiamonds

Fluorescent nanodiamonds (FNDs) with a hydrodynamic diameter of 70 nm were provided by Adámas Nano (Raleigh, NC, USA). In this study, we have used oxygen-terminated and amino-terminated nanodiamonds. They are produced by the manufacturer by grinding high-pressure high-temperature (HPHT) diamonds. According to the vendor, these particles are irradiated with an electron beam at 3 MeV to 5 × 10^19^ e/cm^2^ fluorescence followed by high temperature annealing above 600 °C under vacuum for 2 h.^31^ The NV^–^ content was measured by the manufacturer by electron paramagnetic resonance to be about ∼2–2.5 ppm. This means each particle hosts about 500 centers. The shape and surface chemistry have been rigorously characterized in previous studies.^31,32^

### 4.2 Sperm Preparation

The boar semen was provided by Varkens KI Nederland BV. A Ficoll-based separation technique was used for selection of good-quality spermatozoa. The semen was centrifuged (300*g*, 20 min) over a density gradient of Ficoll-400 (Sigma-Aldrich, Zwijndrecht, The Netherlands), which separates cells by their motility. The discontinuous density gradient of Ficoll-400 was obtained with a 40% (v/v) density top layer and 80% (v/v) density lower layer. The motile spermatozoa formed a soft pellet at the bottom of the tube, collected, and then dispersed in the Ringer rinsing solution. After the second centrifugation (500*g*, 10 min), the pellet was dispersed in noncapacitating (modified Human Tubal Fluid, mHTF, without BSA, NaHCO_3_, and CaCl_2_)^33^ or capacitating medium (Human Tubal Fluid, HTF–NaCl 101.6 mM, KCl 4.69 mM, glucose 2.78 mM, KH_2_PO_4_ 0.37 mM, MgSO_4_ 0.2 mM, sodium lactate 21.4 mM, sodium pyruvate 0.33 mM, BSA 4 mg/mL, NaHCO_3_ 25 mM, CaCl_2_ 2.04 mM).^34^ Sperm cells were counted in a Bürker-Türk chamber under the microscope. The capacitation status was confirmed as described in the Supporting Information (see Figure S3).

### 4.3 Spermatozoa Immobilization and Treatment with FNDs

Sperm cells (1.5 × 10^4^ cells/cm^2^) were immobilized on a glass-bottom Petri dish functionalized with 5 μg/mL fibronectin (Sigma-Aldrich, Zwijndrecht, The Netherlands). After 30 min of incubation at 37 °C, cells were treated with a suspension of nanodiamonds in mHTF medium at concentrations of 1 or 10 μg/mL and kept at 37 °C for 3 h. Afterward, samples were washed with fresh medium to remove any excess of nanodiamonds.

### 4.4 FNDs Location on Sperm Cells

The location of nanodiamonds attached to spermatozoa was determined before and after capacitation. Samples were fixed in 3.7% PFA (paraformaldehyde) and stained for confocal laser scanning microscopy (CLSM) with 2 μg/mL phalloidin-FITC (Sigma-Aldrich, Zwijndrecht, The Netherlands) to label F-actin and 4 μg/mL DAPI to visualize the nucleus (Sigma-Aldrich, Zwijndrecht, The Netherlands). The samples were imaged using 405, 488, and 561 nm lasers of a Zeiss LSM780 confocal microscope. Images were analyzed with FIJI 2.0.0 software. Moreover, the FND distribution was investigated with scanning electron microscopy (SEM). To prepare samples for the SEM observations, a 20 nm layer of gold was deposited on the samples using Leica EM ACE600 magnetron sputter coater. The observations were performed using an FEI Versa 3D FEG scanning electron microscope. The imaging was carried out using an EDT secondary electron detector. An acceleration voltage of 10 kV and a beam current of 4 nA was used.

### 4.5 Viability of FND-Treated Sperm Cells

An MTT assay was performed to evaluate the metabolic activity of sperm cells exposed to the nanodiamonds. This assay detects the activity of NAD(P)H-dependent oxidoreductases, giving a measurement of cell metabolic activity. Cells cultured in 24-well fibronectin-coated plates were treated with 0.75 μg/mL MTT dissolved in mHTF or HTF medium, for uncapacitated or capacitated state, respectively. After 3 h of incubation at 37 °C, the reagent was removed and 2-propanol was added to the samples to dissolve the formazan formed inside the cells. The absorbance of the colored solution was measured using a FLUOstar Omega Microplate Reader (BMG Labtech, De Meern, The Netherlands) at 570 nm. All samples were related to the negative control (immobilized cells untreated with FNDs) after subtraction of the background (medium without cells). Sperm cells treated with the 0.1 M HCl were used as a positive control, while sperm cells without any treatment were used as a negative control. All experiments were performed in triplicates. Results of the MTT assay were validated using a FLUOstar Omega Microplate Reader (BMG Labtech, De Meern, The Netherlands) at 570 nm. Samples were normalized against the mean absorbance value of the negative control, represented as a line at the value 1.

Plasma membrane integrity was determined based on a nucleic acid staining with SYBR-14 (staining of living cells in green) and propidium iodide (PI, staining of cells with damaged membranes in red). The LIVE/DEAD Sperm Viability Kit (Molecular Probes Inc., USA) was used according to the manufacturer’s instructions. Stained cells were visualized using a fluorescent microscope (Zeiss LSM780) with blue (460–490 nm) and green (530–550 nm) excitations for SYBR-14 and PI, respectively. The number of live and dead cells was counted in each sample. Results are presented as a percentage of live cells compared to the control.

### 4.6 T1 Measurements

To study the dynamics of free radical production in real-time during the capitation process, T1 measurements were performed and compared with results of a cellular superoxide detection assay.

For relaxometry experiments, after incubating with FNDs, cells were washed with mHTF medium for the uncapacitated state, and the first T_1_ measurement was performed in a selected FND located on the acrosomal part of the sperm head. After this measurement, a chemical agent (1 μL/ml apocynin (AP), 10 μL/ml progesterone (P), 50 mM diazoxide (DIAZ), or carbonyl cyanide 5 μM CCCP was added to the mHTF medium in order to trigger or inhibit capacitation depending on the experiment. T_1_ measurements followed for 1 h. Finally, mHTF medium was replaced by HTF medium for capacitation and T_1_ was followed for another hour.

The relaxometry experiments (T_1_) were performed using a homemade magnetometry setup described elsewhere,^35^ which in principle is a confocal microscope equipped with a pulsing capacity and an avalanche photodiode (Excelitas, SPCM-AQRH) as a detector. Every T_1_ measurement started with a confocal scan and widefield microscopy. A particle located on the acrosomal part of a living sperm cell was selected during the imaging process for T1 measurements. The viability of the sperm was verified by observing the movement of the cell. Furthermore, we verified that the particle was indeed a diamond particle via its stable fluorescence above 1 million photon counts per second without any bleaching. Particles with counts above 3 million photons were also rejected since these are most likely large aggregates. Once suitable cells and particles were identified, we started the T_1_ measurements. For a T_1_ measurement, the NV^–^ centers were polarized with a laser pulse into the (bright) ms = 0 state of the ground state. Then we measured again after specific dark times to see whether the NV^–^ centers were still in this state or had returned to (dark) equilibrium between ms = 0 and ms = +1 or −1. The time it takes to reach the equilibrium gives a quantitative measurement of the radical concentration in the nanodiamond’s surroundings. In this study, we used 5 μs long laser pulses (532 nm) separated by dark times (*t*) between 200 ns to 10 ms. To obtain a sufficient signal-to-noise ratio, we repeated the pulse sequence 10000 times for each T_1_ measurement. The laser power was set to 50 mW (at the location of the sample) in order to polarize the NV centers without affecting the viability of spermatozoa. The data was analyzed with MatLab software version R2018b.

### 4.7 Cellular Superoxide Detection

Cellular superoxide was detected using the Cellular Superoxide Detection Assay Kit (Abcam, ab139477) following the manufacturer’s protocol. Briefly, sperm cells were plated as described previously. One μM of the superoxide detection compound was added followed by incubation for 1 h at 37 °C. Three time points were evaluated. The first one (0 h) was sperm cells in mHTF medium only; the second one (1 h) was sperm cells after 1 h of treatment with a chemical agent used for T1 measurements (AP, P, CCCP, or DIAZ). And the third time point (2 h) was after 1 h with the respective chemical agent followed by another hour in HTF medium. After staining, cells were washed in their cell culture medium twice and the fluorescent product was visualized using a fluorescence microscope at an excitation of 561 nm and an emission of 620 nm. The fluorescence intensity from heads of sperm cells was measured using Fiji and plotted using Graphpad Prism version 8.

### 4.8 Statistical Analysis

Statistical analysis was performed using Graphpad Prism version 8. Data were treated with a one-way ANOVA and the Wilcoxon test. Significance was tested compared to the uncapacitated sperm and defined as ns *p* > 0.05, *P ≤ 0.05, **P ≤ 0.01, ***P ≤ 0.001, and ****P ≤ 0.0001.

## Supporting Information Available

Capacitated spermatozoa treated with FNDs under SEM (PDF)
Figure S2, video which supports that we measure indeed in living cells as well as imaging results which prove that we have indeed achieved capacitation of sperm cells in the capacitation medium (AVI)

## Supplementary Material

nn2c02511_si_001.pdf

nn2c02511_si_002.avi
