# Supplementary material for: Nanoscale MRI for Selective Labeling and Localized Free Radical Measurements in the Acrosomes of Single Sperm Cells
Source: ACS Nano. 2022 Jun 30;16(7):10701–10. doi: 10.1021/acsnano.2c02511 (PMC9331174; doi:10.1021/acsnano.2c02511)
Supplement: Supplementary file 1 — nn2c02511_si_001.pdf [file nn2c02511_si_001.pdf]

Nanoscale MRI for selective labelling and localised free radical measurements in the acrosomes of single sperm cells

Claudia Reyes-San-Martin[#1], Thamir Hamoh[#1], Yue Zhang[1], Lotte Berendse[1], Carline Klijn[1], Runrun Li[1], Arturo E. Llumbet[1,2], Alina Sigaeva[1], Jakub Kawałko[3], Aldona Mzyk[*1,4], Romana Schirhagl[*1]

[1] Groningen University, University Medical Center Groningen, Antonius Deusinglaan 1, 9713 AW Groningen, The Netherlands

[2] Laboratory of Genomic of Germ Cells, Biomedical Sciences Institute, Faculty of Medicine, University of Chile. Independencia, 1027, Independencia Santiago, Chile

[3] AGH University of Science and Technology, Academic Centre for Materials and Nanotechnology, Al. A. Mickiewicza 30, 30-059 Krakow, Poland

[4] Institute of Metallurgy and Materials Science, Polish Academy of Sciences, Reymonta 25, 30-059 Krakow, Poland

# These two authors contributed equally

*Corresponding author

Supplementary information

Materials and methods

Capacitation status

Sperm cells were evaluated to confirm their capacitation status when incubated on uncapacitating (mHTF) and capacitating (HTF) medium over time. After immobilisation and incubation with 1 µg/ml oxygen terminated FNDs, samples were fixed in 3.7% PFA (Paraformaldehyde) and immunostained for confocal laser scanning microscopy (CLSM). Samples were permeabilised in 0,5% Triton X-100 in PBS for 5 min, followed by 30 min of blocking in 0,5% bovine serum albumin in PBS. Cells were incubated with a mouse-anti-pY antibody incubated for 1 h at room temperature (Sigma-Aldrich P5872; 10 µg/ml). Secondary antibodies goat-anti-mouse IgG H&L conjugated with FITC and DAPI were incubated for 30 min at room temperature. Confocal stack images were acquired in a *Zeiss LSM780* microscope using a 63xW objective. Images were acquired using lasers in the Excitation/Emission range of 358⁄461 nm for DAPI, 488/520 nm for FITC and 532/700 nm for FNDs. A voxel size of 200 x 190 x 190 nm was used.

Results

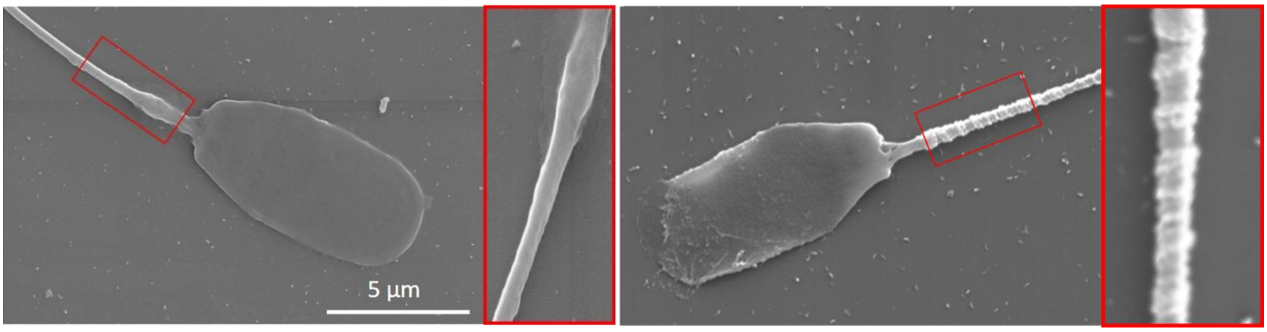

Figure S1 Capacitated spermatozoa treated with FNDs. On the left sperm cell with a smooth mid-piece that seems to do not adhere FNDs, while the sperm cell on the right with helical mid-piece adheres FNDs to the acrosomal part of the head.

Figure S2. Selection of FNDs on a motile sperm cell for T1 experiments. The video shows a timelapse of a motile single sperm cell while T1 measurement is performed. The bright spot on the centre of the head represents the laser focal point where the FND is located.

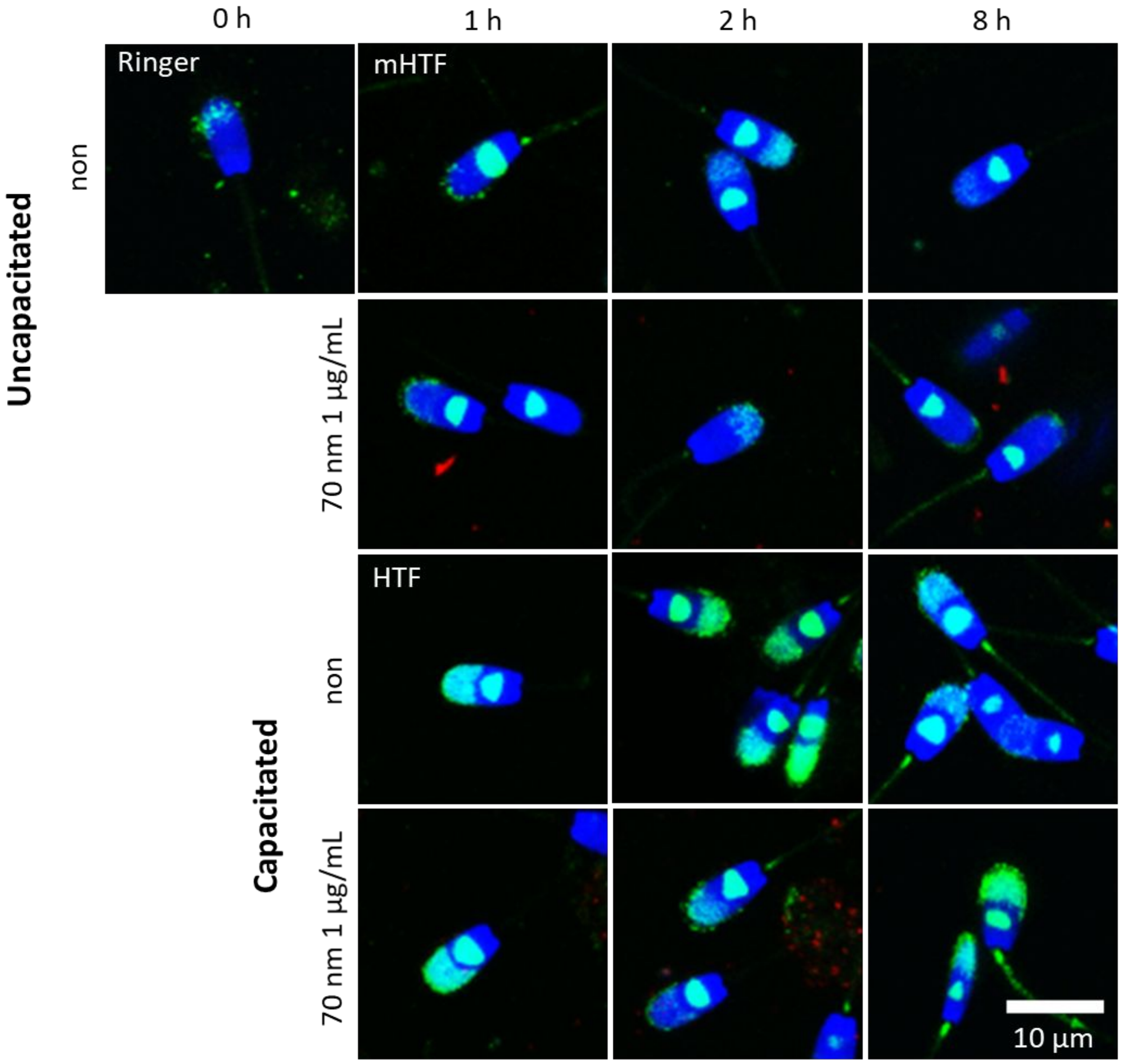

Figure S3. Capacitation status of sperm cells exposed to different cell culture media over time. The staining was performed using immunofluorescence anti-phosphotyrosine protein labelled with FITC (green), In blue, the genetic material labelled with DAPI is shown, and the oxygen terminated FNDs in red. We noticed increased fluorescence intensity on the acrosomal region of cells in HTF medium compared to cells in uncapacitated medium (mHTF or Ringers' solution).
